# Few Associations Found between Mold and Other Allergen Concentrations in the Home versus Skin Sensitivity from Children with Asthma after Hurricane Katrina in the Head-Off Environmental Asthma in Louisiana Study

**DOI:** 10.1155/2012/427358

**Published:** 2012-12-06

**Authors:** L. F. Grimsley, J. Wildfire, M. Lichtveld, S. Kennedy, J. M. El-Dahr, P. C. Chulada, R. Cohn, H. Mitchell, E. Thornton, M. Mvula, Y. Sterling, W. Martin, K. Stephens, L. White

**Affiliations:** ^1^School of Public Health and Tropical Medicine, Tulane University, New Orleans, 1440 Canal Street, Suite 2100 (SL-29), New Orleans, LA 70112, USA; ^2^Rho, Chapel Hill, NC 27517, USA; ^3^Department of Pediatrics, School of Medicine, Tulane University, New Orleans, LA 70112, USA; ^4^Program in Clinical Research, National Institute of Environmental Health Sciences, Research Triangle Park, 27709, USA; ^5^SRA International Incorporation, Durham, NC 27713, USA; ^6^Visionary Consulting Partners, LLC, Fairfax, VA 22030-3409, USA; ^7^Division of Health Disparities, New Orleans Health Department, New Orleans, LA 70112, USA; ^8^Health Sciences Center School of Nursing, Louisiana State University, New Orleans, LA 70112, USA; ^9^National Institute of Child Health and Human Development, Bethesda, MD 20892, USA

## Abstract

Mold and other allergen exposures exacerbate asthma symptoms in sensitized individuals. We evaluated allergen concentrations, skin test sensitivities, and asthma morbidity for 182 children, aged 4–12 years, with moderate to severe asthma, enrolled 18 months after Katrina, from the city of New Orleans and the surrounding parishes that were impacted by the storm, into the Head-off Environmental Asthma in Louisiana (HEAL) observational study. Dust (indoor) and air (indoor and outdoor) samples were collected at baseline of 6 and 12 months. Dust samples were evaluated for dust mite, cockroach, mouse, and *Alternaria* by immunoassay. Air samples were evaluated for airborne mold spore concentrations. Overall, 89% of the children tested positive to ≥1 indoor allergen, with allergen-specific sensitivities ranging from 18% to 67%. Allergen concentration was associated with skin sensitivity for 1 of 10 environmental triggers analyzed (cat). Asthma symptom days did not differ with skin test sensitivity, and surprisingly, increased symptoms were observed in children whose baseline indoor airborne mold concentrations were below median levels. This association was not observed in follow-up assessments. The lack of relationship among allergen levels (including mold), sensitivities, and asthma symptoms points to the complexity of attempting to assess these associations during rapidly changing social and environmental conditions.

## 1. Introduction

As a result of Hurricane Katrina, much of the city of New Orleans was inundated with water [1]. Approximately 80% of the city was flooded, and this water took more than a month to clear from some areas [2]. These flooded homes created the ideal conditions for mold growth, and there was a concern that high levels of mold would cause an increase in respiratory disease and asthma exacerbations [3]. 

According to the Centers for Disease Control and Prevention (CDC), approximately 9.6 million children (13.1 %) in the United States have been diagnosed with asthma during their lifetimes. The costs of healthcare and lost productivity are estimated to be over 20 billion dollars per year nationwide, and lack of proper treatment and management can result in morbidity and even mortality [4]. Studies have shown that children exposed to high levels of indoor allergens can become sensitized to these allergens and that asthma can be exacerbated by these exposures [5–8]. The purpose of this report is to describe the impact of Hurricane Katrina on environmental exposures, such as mold and other indoor allergens, and their effect on asthma morbidity on children in the Head-off Environmental Asthma in Louisiana (HEAL) study. HEAL began 18 months after Katrina, studying children with asthma from the city of New Orleans and surrounding parishes (NO) that were impacted by the storm.

## 2. Materials and Methods

HEAL was an observational study in which 182 children from NO were enrolled between March 2007 and March 2008. The study design and methods, approved by the National Institute of Environmental Health Sciences and the Tulane and Louisiana State Universities' Institutional Review Boards, are described in detail elsewhere [9]. The objective of the study was to investigate the relationships between childhood asthma morbidity and asthma triggers, including environmental exposures, in a postdisaster setting. Another objective was to intervene in childhood asthma morbidity by implementing and assessing a novel asthma counselor (AC) intervention that provided both asthma case management and guidance for reducing exposure to environmental asthma triggers, including moisture, mold, and other allergens [10]. The intervention drew upon previous studies, including the National Cooperative Inner-City Asthma Study (NCICAS) [6, 11, 12] and the Inner-City Asthma Study (ICAS) [8, 13] but was designed to meet the needs of individuals living in post-Katrina environments.

In brief, children were recruited from NO schools and prescreened by telephone. They were eligible if they were 4–12 years old, lived in Orleans Parish or neighboring parishes that were affected by Katrina, had been diagnosed with asthma by a physician, and met criteria for moderate-to-severe asthma as assessed during this prescreening phone interview [14]. They also had to sleep in the same home ≥5 nights/week, have no plans to move from the area during the year, and have a legal guardian who had access to a phone as their caregiver. This prescreening eligibility questionnaire was ended at the first point where a potential participant was deemed ineligible, and additional data (e.g., demographics, exposures) was not collected for those who were ineligible. Enrolled participants received 2 clinical evaluations, 3 home evaluations, 4 quarterly outcomes phone evaluations, and at least 2 asthma counseling visits performed over a 1-year period. 

At the baseline clinical evaluation, informed consent was obtained from caregivers, and assent was obtained from children. Afterward, the children underwent an extensive clinical evaluation to confirm eligibility and collect baseline data using survey instruments adopted from NCICAS and ICAS and modified for HEAL [9]. Procedures included the collection of medical histories (emphasizing respiratory symptoms and asthma medications), pulmonary function testing, and blood assays. Allergen skin testing was performed on the volar surface of the children's arms using a multitest device (Lincoln Diagnostics, Inc., Multitest II). The panel consisted of standard indoor allergens, including dust mites (Der p. and Der f. mix), cockroach mix (American and German), cat pelt, dog pelt, mouse, rat, and molds (*Alternaria*, *Cladosporium*, *Aspergillus fumigatus*, and *Penicillium chrysogenum (notatum)*), and Bermuda grass. Additional skin testing was performed for 10 other prevalent molds found in NO (*Neurospora, Acremonium, Chaetomium, Drechslera, Epicoccum, Paecilomyces, Trichoderma, Bipolaris, Fusarium, *and* Aspergillus niger*) [15, 16] for a total of 14 molds tested. A skin test was considered positive, if the allergen wheal was at least 3 mm greater than the negative control. The 12-month clinical evaluation included, similar procedures, but allergen skin testing was not performed. 

The homes of HEAL children were evaluated by 2 trained environmental assessors within 3 weeks of the baseline clinical evaluation and again 6 and 12 months later. These evaluations consisted of indoor visual inspections, indoor dust and air sampling, and outdoor air sampling. During the indoor inspection, assessors looked for signs of moisture and water damage, environmental tobacco smoke, pest infestation (cockroaches or rodents), mold, and other allergens and environmental hazards. Dust samples were collected from the child's bedroom floor and bed using standardized methods [13]. Bed dust was analyzed for the presence of German cockroach (Bla g 1), dust mite (Der p 1), mouse (Mus m 1), and *Alternaria *(Alt a 1) [17]. Samples with insufficient quantities of bed dust were supplemented or replaced with bedroom floor dust. The remaining dust from the bed or floor was then analyzed for endotoxin and 1→3- and 1→6-*β*-D-glucan levels using the Limulus Amebocyte Lysate Kinetic-QCL Test Kit and an enzyme-linked immunosorbent assay, respectively [18]. Air samples were collected from the child's sleeping area/bedroom and outside the home based on previously validated methods [19] using calibrated high airflow pumps and Air-O-Cell spore traps (Zefon International, Ocala, FL, USA). Air samples were analyzed for mold spore count [17].

Outcome measures were collected using an asthma symptoms and healthcare utilization survey that was administered during the clinical evaluations and quarterly outcome calls. The primary outcome was the number of self-reported maximum symptoms days (MSD) for asthma in the preceding 2 weeks. This value was defined as the largest number of the following variables: days with wheezing, chest tightness, or cough (daytime symptoms); nights of sleep disturbance (night symptoms); days when activities were affected (slow play), such as the child needing to slow down or end physical activities. Secondary outcomes included the individual symptom variables listed above and disruptions in the caretaker's schedule because of the participant's asthma (caretaker changed plans). The rates of sensitivity for high/low allergen concentrations were compared using chi-square tests. For the exploratory analysis of the morbidity data, HEAL children were divided into 4 groups based on allergen concentrations and skin test sensitivity (above median concentration and positive sensitivity; above median concentration and negative sensitivity; below median concentration and positive sensitivity; below median concentration and negative sensitivity). Next, average morbidity levels (primary and secondary outcomes) were calculated for each group and compared using a general linear model with an interaction term between allergen concentration and sensitivity. This model indicates whether symptoms changed significantly across different levels of sensitization and allergen concentration. A *P* value of 0.10 was considered significant [20]. Analyses were conducted using SAS version 9.2 (Cary, NC, USA) and R version 2.13.0.

## 3. Results 

The baseline characteristic of HEAL children are presented in Table 1. Many HEAL children (37%) lived in homes that flooded during Katrina. Bedroom airborne mold allergen concentrations averaged 501 spores/m^3^, and bedroom dust-borne *Alternaria* levels averaged above 10 *μ*g/g 58% of the time. Indoor allergens other than *Alternaria* were detected in 75% of the homes. The majority of children (89%) had at least one positive skin test response, and individual sensitivities ranged from 18% (children sensitive to rats) to 67% (children sensitive to dust mites).

The relationships between environmental allergen concentrations and skin sensitivities are shown in Table 2. A relationship was observed between cat ownership and sensitivity; children with a cat (63%) were more likely to have a positive cat skin test than children without a cat (33%, *P* = 0.03). However, no relationships were observed between skin sensitivity and allergen concentrations for dust-borne cockroach, dust mite, mouse, *Alternaria*, dog, *β*-glucan, or endotoxin (all *P* > 0.15). Likewise, no relationships were observed between airborne mold (indoor or outdoor) concentrations and mold skin sensitivity (Table 2). 

The relationships among environmental allergen levels, skin test sensitivity, and asthma morbidity are shown for dust allergens (Table 3) and airborne mold (Table 4). MSD, the primary outcome, did not differ among allergen concentration/sensitivity groups for dust mite, cockroach, mouse, or *Alternaria* (all *P* > 0.15). Similar results were observed for the secondary outcomes. While there was a significant increase in days of slow play for children who had a positive skin test for cockroach *and* cockroach allergen above the median concentration (mean of 6.18 days versus 2.85 days for others, *P* = 0.01), this trend was not seen for other outcomes or with other allergens. 

Neither MSD nor the secondary outcomes varied with allergen concentration/sensitivity groups for outdoor airborne mold, *β*-glucan, or endotoxin (all *P* > 0.15). The only significant relationship observed was with indoor airborne mold, but surprisingly, symptoms were significantly higher in children exposed to below-median levels of indoor airborne mold (Table 4). The trend was consistent for both MSD (*P* < 0.01) and several secondary outcomes, including daytime symptoms (*P* < 0.01), days of slow play (*P* = 0.03), night symptoms, (*P* = 0.06), and caretaker changed plans (*P* = 0.05). This inverse symptom-mold relationship appears to have been driven by mold concentration and not sensitivity in HEAL. There is no evidence of a difference in symptom level associated with mold sensitivity alone (all *P* > 0.25, data not shown), but symptoms consistently have an inverse relationship with indoor airborne mold concentration (*P* < 0.001 for MSD; *P* < 0.02 for all secondary outcomes). The inverse relationship between baseline MSD and indoor airborne mold is still present in a model adjusting for parish, season, income, race, age, and sex (*P* < 0.001).

The relationship between indoor airborne mold and MSD is not found at later study visits; there are no significant relationships between unadjusted followup MSD (measured at 6 or 12 months) and airborne mold level (measured at baseline, 6 or 12 months; all *P* > 0.30) (Table 5). Similar nonsignificant results from 6- and 12-month measures are seen after adjustment for the timing of the study intervention, parish, season, income, race, age, and gender (data not shown). 

## 4. Discussion

Hurricane Katrina was the most economically destructive storm in U.S. history [21]. The loss of life and property damage were exacerbated by the levee breaks; at least 80% of New Orleans was reported to be flooded 2 days after the hurricane struck, with some areas having as much as 20 feet of flood water [1]. Public officials and citizens of New Orleans expressed concern that the resulting high levels of mold and other toxic environmental exposures could aggravate existing respiratory disease and cause additional deleterious health effects [22]. These concerns were especially great for those children with asthma in New Orleans, where some of the country's highest rates of this disease have long been documented [23, 24]. The HEAL project measured the environmental allergen concentrations in post-Katrina New Orleans within the homes of children with asthma and provided support for their healthcare through environmental and AC interventions that were shown to be effective in the inner-city environment. 

The environmental impact of Hurricane Katrina was devastating, so much that 94% of the families enrolled in the HEAL project moved at least once following the disaster, and many moved more than 2 or even 4 times to find acceptable housing. Remediation of the Katrina damage began shortly after the hurricane and continued for years. During this study, the environmental conditions improved rapidly, with 68% of the study participants initiating renovations that continued throughout the HEAL study period. In fact, many HEAL children and their caregivers had moved to new residences (49%) or completed home renovations (47%) before enrollment in HEAL. While HEAL children and caregivers moved and rebuilt, airborne mold declined from the peak levels that occurred immediately after the storm. The average airborne mold levels measured at HEAL baseline were markedly lower (501 spores/m^3^ indoors) than measurements taken immediately after Katrina (11,000–645,000 spores/m^3^ indoors) [15, 16, 25]. 

The development of allergen sensitivities can begin at a very early age and, therefore, is difficult to link to short-term exposures, especially those later in life. Increased exposures to allergenic risk factors, such as allergens and molds, can dramatically increase asthma morbidity [26]. Mold skin test sensitivities in HEAL children were significantly higher than those observed in other inner city populations [8], but we were not able to detect a significant difference in asthma symptoms or healthcare utilization in this cohort that was followed a few years after Hurricane Katrina.

Levels of common household dust allergens, with the exception of mold, were lower in HEAL homes than the levels reported in comparable inner-city studies. For example, 20% of the homes of HEAL children had detectable levels of cockroach (Bla g 1) in their bedrooms, and only 4% had levels greater than 2 U/g, which is considered the threshold for skin sensitization [27]. In comparison, 32% of ICAS children had detectable levels of cockroach in their bedrooms, and 12% had levels above 2 U/g [8]. Similarly, HEAL dust allergen levels were lower than comparable dust allergen measurements taken pre-Katrina (20% versus 57% >2 U/g cockroach; 9% vs. 46% >2 *μ*g/g Der p 1) [28]. Clearly, moving to a cleaner home combined with the measures taken to clean damaged HEAL homes led to the lower levels that were observed and the apparent disconnect between post-Katrina allergen levels and observed sensitization rates. While we do have preremediation/prerenovation dust allergen data from some HEAL homes (those who remediated and renovated during HEAL), the number of such homes was too few to make any meaningful comparisons. It is possible that HEAL children were exposed to high allergen levels in locations other than their homes, such as schools or homes of relatives or friends, causing them to become sensitized; however, environmental sampling for HEAL was not conducted in locations other than the children's homes. Also, it is possible that results could have been impacted when bed dust samples were supplemented or replaced with bedroom floor samples because the quantity of bed dust was insufficient for analysis; however, low levels of dust allergens were found across all of the samples regardless of if they were strictly collected from the bed or combined or replaced with bedroom floor samples. The low allergen levels found in the bed/bedroom dust of HEAL children allowed little opportunity to differentiate between “high” and “low” concentrations or to test for relationships with sensitivity. 

As previously mentioned, unlike the other dust allergen concentrations, *Alternaria* dust allergen was found in the majority of all bedrooms (98%) and found at concentrations >10 *μ*g/g in 58% of bedrooms. This finding requires further investigation; in addition, potential implications have been previously discussed [17], including the most likely reason being that this may not only just represent *Alternaria* but may be an artifact from the polyclonal antibody used to detect *Alternaria* cross-reacting with other fungi, such as those belonging to the Pleosporaceae family (*Alternaria, Ulocladium, Stemphylium*) and other dematiaceous genera including *Epicoccum *[29].

For asthma morbidity, previous studies have shown that cockroach, dust mite, and cat exposure are linked to asthma symptoms in sensitized individuals [26, 27, 30]. Exacerbations have also been documented with dog, mouse, and *Alternaria* allergen exposures [31–33]. In HEAL, no similar positive relationships were observed between asthma symptoms and the allergen concentration/sensitivity groups. In the interesting case of airborne mold and morbidity, a robust inverse relationship was observed at baseline; however, this same inverse finding was not seen during followup measures at 6 and 12 months, so a causal relationship is unlikely. While mold levels were high shortly after Hurricane Katrina [15], research less than 1 year after Katrina found that mold levels and respiratory symptoms had decreased and that mold levels in homes with hurricane damage had returned to moderate levels [34]. These findings are consistent with the modest environmental allergen levels observed in HEAL and support the notion that residents renovated their homes or moved to cleaner environments upon returning to NO. This rapid change from a flooded, high-mold environment to a relatively clean environment somewhat limits the clinical utility of the single exposure measurements taken 18 months after Katrina. The low level of environmental allergen concentrations is the most obvious reason for the lack of significant relationships between asthma morbidity and the airborne mold concentrations, dust-borne allergen levels, and skin test sensitivities observed in HEAL. It would be interesting to prospectively reexamine these relationships in HEAL children to see if environmental allergen concentrations increase as the disaster conditions fully stabilize and the renovated homes return to their pre-Katrina state. 

## 5. Conclusion

Overall, several children in the HEAL study tested positive to at least one indoor allergen. Asthma symptom days did not differ with skin test sensitivity, and surprisingly, increased symptoms were observed in children whose baseline indoor airborne mold concentrations were below median levels. However, this association was not observed in followup assessments. Future research should focus on evaluating indoor environmental conditions to determine if there is a change in mold and other allergen concentrations and asthma morbidity in New Orleans post-Katrina.

## Figures and Tables

**Table 1:** Baseline characteristics of the study population (*N* = 182).

Demographics	
Male	98/182 (54%)
Race/ethnicity	
African-American	122/182 (67%)
Caucasian	48/182 (26%)
Hispanic	12/182 (7%)
Household income < $15,000	43/170 (25%)
At least one smoker in the household	58/182 (32%)
Caretaker married	97/182 (54%)
Caretaker completed high school	159/182 (88%)

Housing	

Baseline home is same as home pre-Katrina	92/182 (51%)
Moved at least once between Katrina and baseline	171/182 (94%)
Moved at least once during the study	37/182 (20%)
Baseline home flooded	68/182 (37%)
Completed home renovations at baseline	85/182 (47%)
Renovations ongoing at baseline	38/182 (21%)

Baseline asthma symptoms in the previous 2 weeks	

Maximum symptom days	6.6 ± 4.9
Daytime symptoms	5.3 ± 4.5
Night symptoms	3.3 ± 4.2
Slow play	3.2 ± 3.9
Caretaker changed plans	0.9 ± 1.7

Allergen sensitivities	

Skin tests (≥3 mm wheal)	
Der f/der p mix	121/180 (67%)
Cat	64/180 (36%)
Cockroach mix	93/180 (52%)
Dog	51/180 (28%)
Rat	33/180 (18%)
Mouse	54/180 (30%)
*Cladosporium *	52/180 (29%)
*Aspergillus fumigatus *	43/180 (24%)
*Penicillium chrysogenum (notatum) *	86/180 (48%)
*Alternaria *	96/180 (53%)
Number of mold sensitivities (14 total molds tested)	5.3 ± 5.1

Household allergens	

*Alternaria *	
Detectable	178/181 (98%)
>10 *μ*g/g	105/181 (58%)
Cockroach (Bla g 1)	
Detectable	37/181 (20%)
>2 U/g	8/181 (4%)
Dust mite (Der p 1)	
Detectable	63/181 (35%)
>2 *μ*g/g	16/181 (9%)
Mouse (Mus m 1)	
Detectable	108/181 (60%)
>1.6 *μ*g/g	5/181 (3%)
Microbial component	
*β*-glucan (*μ*g/g)	0.50 ± 0.06
Endotoxin (EU/mg)	11.4 ± 1.5
Self-report of pet ownership	
Dog	46/182 (25%)
Cat	16/182 (9%)

Mold air sampling (spores/m^3^)	

Indoor total	501 ± 48
Outdoor total	3960 ± 467

Values are the count (percent) or mean ± standard deviation. Geometric means are reported for mold air samples.

**Table 2:** Relationship of environmental allergen concentrations to allergen sensitization.

Allergen	Concentration^a^	*N *	Sensitivity^b^	*P* value
Cockroach	Above median	37	47%	0.71
	Below median	144	52%	
dust mite	Above median	63	60%	0.17
	Below median	118	71%	
mouse	Above median	81	36%	0.15
	Below median	100	25%	
*Alternaria *	Above median	90	58%	0.32
	Below median	91	49%	
Cat	Yes	16	63%	0.04
	No	166	33%	
dog	Yes	46	33%	0.58
	No	136	27%	
indoor mold	Above median	90	49%	0.55
	Below median	92	54%	
outdoor mold	Above median	91	54%	0.55
	Below median	91	49%	
endotoxin	Above median	69	51%	0.81
	Below median	71	55%	
*β*-Glucan	Above median	54	48%	0.29
	Below median	55	60%	

^a^Median concentrations: cockroach—0.2 U/g (lower limit of detection), dust mite—0.1 *μ*g/g (LLOD), mouse—0.03 *μ*g/g (LLOD), *Alternaria*—13.7 *μ*g/g (LLOD), indoor mold—514 spores/m^3^, outdoor mold—3840 spores/m^3^, endotoxin—12 EU/mg, *β*-Glucan—0.4 *μ*g/g. Cat and dog exposure were determined via a yes/no questionnaire item.

^b^For indoor allergens (cockroach, dust mite, mouse, *Alternaria*, cat, and dog), sensitivity is indicated by a positive skin test to the given allergen (wheal at least 3 mm greater than the negative control). For mold, endotoxin, and *β*-Glucan, sensitivity is indicated by 4 or more positive skin tests out of 14 mold skin tests measured.

**Table 3:** Relationship among asthma morbidity, dust allergen sensitivity, and dust allergen concentration.

Outcome^a^	Concentration^b^	Sensitivity^c^	Allergen							
			Cockroach	*P *	Dust mite	*P *	Mouse	*P *	*Alternaria *	*P *
*N *	Above median	Positive	17		37		29		51	
		Negative	19		25		51		37	
	Below median	Positive	75		83		25		45	
		Negative	68		34		74		46	

Maximum symptom days	Above median	Positive	8.24		7.38		7.14		7.82	0.16
		Negative	6.42	0.37	6.80	0.44	6.33	0.56	6.78	
	Below median	Positive	6.89		6.72		5.60		5.71	
		Negative	6.03		5.53		7.01		6.13	

Daytime symptoms	Above median	Positive	5.82		5.89		5.34		6.35	
		Negative	5.42	0.98	5.40	0.57	5.22	0.96	5.22	0.34
	Below median	Positive	5.37		5.55		5.16		4.80	
		Negative	5.29		4.44		5.61		5.04	

Night symptoms	Above median	Positive	2.94		3.62		4.86		4.25	
		Negative	2.79	0.33	2.48	0.51	3.06	0.19	2.30	0.18
	Below median	Positive	3.99		3.64		3.00		3.11	
		Negative	2.78		2.74		2.96		3.24	

Slow play	Above median	Positive	6.18		3.81		4.34		3.84	
		Negative	2.89	0.01	2.88	0.39	2.63	0.16	2.92	0.54
	Below median	Positive	3.13		3.34		2.28		2.98	
		Negative	2.54		2.29		3.39		2.83	

Caretaker changed plans	Above median	Positive	1.53		0.81		1.34		1.12	
		Negative	0.95	0.33	0.80	0.97	0.98	0.21	0.43	0.27
	Below median	Positive	0.71		0.95		0.40		1.00	
		Negative	0.93		0.91		0.82		0.91	

^a^Outcome units are “number of days in the past 2 weeks.” *P* values test for an interaction between allergen sensitivity and concentration for the given outcome.

^b^Median concentrations: cockroach—0.2 U/g (lower limit of detection), dust mite—0.1 *μ*g/g (LLOD), mouse—0.03 *μ*g/g (LLOD), *Alternaria*—13.7 *μ*g/g (LLOD).

^c^Skin test wheal was at least 3 mm greater than the negative control; allergen sensitivity corresponds to dust allergen exposure (e.g., cockroach allergen sensitivity and concentration).

**Table 4:** Relationship among asthma morbidity, mold sensitivity and airborne mold, endotoxin, or glucan concentrations.

Outcome^a^	Concentration^b^	Sensitivity^c^	Allergen							
			Indoor mold	*P *	Outdoor mold	*P *	Endotoxin	*P *	*β*-Glucan	*P *
*N *	Above median	Positive	44		49		35		26	
		Negative	46		41		33		28	
	Below median	Positive	49		44		39		33	
		Negative	41		46		32		22	

Maximum symptom days	Above median	Positive	5.39		6.20		7.14		6.38	
		Negative	5.12	<0.01	6.63	0.54	7.21	0.75	6.50	0.96
	Below median	Positive	8.10		7.50		7.62		7.00	
		Negative	7.85		6.20		6.31		6.45	

Daytime symptoms	Above median	Positive	4.02		5.22		6.11		5.92	
		Negative	4.07	<0.01	5.32	0.91	5.55	0.97	4.93	0.89
	Below median	Positive	6.82		5.80		6.03		5.52	
		Negative	6.51		5.13		5.91		5.41	

Night symptoms	Above median	Positive	3.05		3.51		3.63		4.15	
		Negative	2.15	0.06	2.98	0.33	2.82	0.50	2.75	0.43
	Below median	Positive	4.47		4.11		4.38		3.42	
		Negative	3.44		2.57		3.38		2.36	

Slow play	Above median	Positive	2.82		3.04		3.37		2.77	
		Negative	1.98	0.03	2.54	0.23	3.30	0.17	2.61	0.97
	Below median	Positive	4.24		4.16		3.92		2.91	
		Negative	3.54		2.87		1.94		3.05	

Caretaker changed plans	Above median	Positive	0.70		1.00		1.20		1.23	
		Negative	0.41	0.05	0.80	0.90	0.67	0.44	0.50	0.42
	Below median	Positive	1.22		1.00		0.72		0.79	
		Negative	1.22		0.78		1.13		0.95	

^a^Units are “number of days in the past 2 weeks.” *P* values test for an interaction between allergen sensitivity and concentration for the given outcome.

^b^Median concentrations: indoor mold—514 spores/m^3^, outdoor mold—3840 spores/m^3^, endotoxin—12 EU/mg, *β*-Glucan—0.4 *μ*g/g.

^c^Four or more skin test wheals that are at least 3 mm greater than the negative control out of 14 mold skin tests measured.

**Table 5:** Relationship between airborne mold levels and asthma morbidity by study visit.

Home evaluation	Symptom assessment	Number of maximum symptom days			*P* value
		Airborne mold > median	Airborne mold ≤ median	Diff
Baseline	Baseline	5.2	8.0	−2.8	<0.001
	6 months	3.2	3.5	−0.3	0.65
	12 months	3.4	3.8	−0.4	0.56
6 months	6 months	3.1	3.0	−0.1	0.80
	12 months	3.6	3.6	−0.0	0.94
12 months	12 months	3.4	3.5	−0.1	0.89

Values are unadjusted for potential confounding variables (e.g., parish, season, income, race, age, and sex). Followup MSD were measured at 6 or 12 months and airborne mold levels were measured at baseline 6 or 12 months.
